# Enzymatic synthesis of bio-based polyesters derived from vanillin

**DOI:** 10.3389/fchem.2026.1769290

**Published:** 2026-02-20

**Authors:** Cicely M. Warne, Maria Jose Calandri, Myleidi Vera, Georg M. Guebitz, Alessandro Pellis

**Affiliations:** 1 ACIB GmbH, Tulln ander Donau, Austria; 2 Department of Agricultural Sciences, Institute of Environmental Biotechnology, BOKU University, Tulln ander Donau, Austria; 3 Department of Chemistry and Industrial Chemistry, University of Genova, Genova(GE), Italy; 4 Departamento de Polímeros, Facultad de Ciencias Químicas, Universidad de Concepción, Concepción, Chile

**Keywords:** bio-based polymers, biocatalysed synthesis, enzymatic polycondensation, green solvents, lipase, vanillin-derived polyesters

## Abstract

Aromatic polymers are known for their thermal stability as well as their good mechanical properties, but most of these polymers are synthesised from fossil resources. Vanillin is one of the few aromatic chemicals that is currently commercially produced from biomass and can be derivatised to make it suitable for polycondensation reactions. In this work a vanillin-derived bio-based diol was synthesised exploiting the potential of more benign reagents to replace toxic dimethylformamide. The resulting monomer was utilised in a series of enzymatic polycondensation reactions with different diesters including dimethyl succinate (DMSu), dimethyl adipate and dimethyl sebacate (DMSe), and the aromatic monomers diethyl terephthalate diethyl isophthalate diethyl pyridine-2,5-dicarboxylate (PD25) and diethyl pyridine-2,4-dicarboxylate (PD24) using a lipase to produce semi-aromatic polyesters. The molecular weight of the resulting polyesters increased as the chain length of the diester decreased, with a number average molecular weight of 21.8 kDa for polyesters achieved based on the dimethyl succinate (using diphenyl ether (DPE) as the organic media). For semi-aromatic diesters, *para-*substituted monomers yielded higher molecular weight polymers compared to the corresponding *meta-*substituted structure. Several different green solvents were also investigated to carry out this reaction with anisole that resulted to be a good alternative to diphenyl ether with similar number average molecular weights obtained at certain conditions.

## 1 Introduction

Plastics containing aromatic moieties tend towards having good mechanical properties, thermal resistance and gas barrier properties. Indeed, the aromatic polymers polybenzimidazole (PBI) and poly (ether ketone) (PEEK) are well known for their high thermal stability and excellent tensile strength (McKeen, 2010), and the toughness, transparency and relatively easy recyclability of poly (ethylene terephthalate) (PET) make it a popular choice for food packaging materials (Nisticò, 2020). These plastics are however, almost entirely petroleum-based and therefore unsustainable from an environmental point of view. Although the terephthalate moiety in PET can be bio-derived, it is currently prohibitively expensive compared to fossil-derived terephthalic acid (Carus et al., 2014). Instead, polymers such as poly (ethylene furanoate) (PEF), which are more easily synthesised from biomass are steadily gaining interest as PET replacements (Prasad et al., 2023). Despite the increasing popularity of PEF, the range of aromatic bio-based plastics is currently limited, and other commercially available aliphatic bioplastics such as PLA and PHA have noted poor thermostability (Kervran et al., 2022). To fully replace petroleum derived plastics, a wider range of bio-based aromatic plastics are needed, and this requires developing novel bio-based aromatic monomers.

Instead of using chemistry to add aromaticity to these building blocks, focus has instead turned towards naturally occurring aromatic compounds, namely, produced from lignin. This is the second most abundant biopolymer on earth, with an estimated 50–70 MT per year produced as a by-product from the pulp and paper industry (Mandlekar et al., 2018). Despite this, it is incredibly under-utilised, with less than 2% of waste lignin used to produce specialty chemicals (Mandlekar et al., 2018). This is due to the unique challenges of this polymer; its non-uniform structure and recalcitrance towards both biological and chemical treatments make it difficult to valorize (Sethupathy et al., 2022). There is also considerable variation within the lignin itself depending on origin and method of extraction (Chio et al., 2019), which adds a further layer of complexity.

Perhaps the most successful lignin-derived compound is vanillin, which can be produced from lignin via aerobic oxidation in alkaline media (Schutyser et al., 2018). This method has fallen out of favor and is now solely used by the Norwegian company Borregaard (Borregaard, 2024), with the vast majority of vanillin on the market instead synthesised from petroleum-sourced phenol in a two-step process (Ciriminna et al., 2019). Despite this, there exist more sustainable routes to vanillin from lignin, such as electrochemical depolymerization, oxidative depolymerization as well as biotransformation using microbes or enzymes (D’Arrigo et al., 2024). As one of the few industrially available aromatic products from lignin, vanillin has been used as a building block in the synthesis of many aromatic polymers.

Vanillin is an extremely versatile compound, having been used to synthesise both thermoplastics and thermosets. One example are epoxy resins; a common type of thermosetting polymer typically synthesised from fossil-derived bisphenol A (BPA), which can instead be produced from vanillin derivatives (Su et al., 2020; Wang et al., 2017). Vinyl and cyanate ester resins have also been synthesised using vanillin as a starting material (Harvey et al., 2015; Zhang, 2022b). Thermoplastics can also be synthesised from vanillin, and there are many examples of polyurethanes, polycarbonates, polyolefins and polymethacrylates in recent literature (Holmberg et al., 2014; Llevot, 2015a; Harvey et al., 2015; Bai et al., 2018; Fanjul-Mosteirín et al., 2023). Vanillin-based polyesters have been noted for their exceptional thermal and mechanical properties in particular, and polyesters in general have the advantage of recyclability when compared to polymers without hydrolysable bonds such as polyolefins. Llevot *et al.* synthesised a methylated divanillyl diol and a methylated dimethylvanillate dimer and used them to produce semi aromatic polyesters with a thermal stability of up to 350 °C (Llevot, 2015b). Vanillin has also been used to produce the diols 4-(hydroxymethyl)-2-methoxyphenol and 2-(4-(hydroxymethyl)-2-methoxyphenoxy) ethanol, and polymerisation of these monomers with acyl chlorides resulted in polyesters with a high glass transition temperature (T_g_) (Zhao et al., 2020). Xanthopoulou *et al.* synthesised poly (hexylene vanillate) which had remarkable elastic recovery and was thermally stable up to 385 °C (Xanthopoulou et al., 2023). Mialon *et al.* produced acetyldihydroferulic acid from vanillin in a two-step process which could then be directly polymerised. The polymer produced had similar thermal characteristics to PET, with a lower melting temperature (T_m_) which is useful for processing (Mialon et al., 2010). The same group also produced several aromatic-aliphatic vanillin-based polyesters with varied aliphatic chain lengths, and showed that T_g_ decreases with increasing number of carbons in the aliphatic chain (Mialon et al., 2011). A point of similarity between these works is the modification of vanillin before polymerisation; this compound can be derivatised in many different ways to obtain functionalities desired in polymers. It is also possible to synthesise monomers suitable for polycondensation with alternative catalysts, such as enzymes.

Biocatalysts are green and sustainable catalysts, characterised by their bio-based nature, non-toxic and preference for mild conditions. *Candida antarctica* Lipase B (CaLB) is tolerant of aromatic substrates, having been used to synthesise polyesters from bio-based aromatic compounds such as pyridine based diesters (Pellis et al., 2019), and dimethyl 2,5-furandicarboxylate (Jiang et al., 2015). One limitation of this enzyme is its lack of reactivity for phenolic functional groups, generally because they are far less nucleophilic. Phenolic compounds such as vanillin therefore require modification of phenol groups before they can be enzymatically polymerised, which can be done through reaction with a carbonate, resulting in primary hydroxyl groups (Mankar et al., 2019).

**FIGURE 1:**
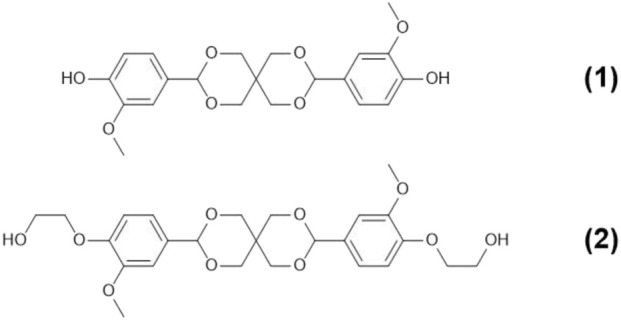
The structure of the vanillin-based Spiro-bisphenol (Compound **(1)**) and Spiro-diol V (Compound **(2)**) developed by Mankar et al. (2019).

This paper investigates the use of CaLB to synthesise several semi-aromatic polyesters from vanillin derived monomers. The compound Spiro-diol V (Figure 1) from the work of Mankar et al. (2019), hereafter referred to as Compound **(2)**, was selected as a structure that has the potential to be polymerised via biocatalysis. This can be readily synthesised via a two step acetalization of vanillin with pentaerythritol resulting in Compound **(1)** (Figure 1), followed by ethylation with ethylene carbonate to form Compound **(2)** (Mankar et al., 2019). This is a relatively green procedure, and the introduction of spirocyclic units into a polymer should improve its recyclability (Valsange et al., 2024).

## 2 Materials and methods

### 2.1 Materials

Sodium hydroxide (BioXtra, ≥98% (acidimetric), pellets (anhydrous)), Diphenyl ether (ReagentPlus®, ≥99%), Cyrene™ (≥98.5%), Dimethyl sebacate (99%), Dimethyl succinate (98%), *Candida antarctica* Lipase B (CaLB, code: L4777), chloroform (suitable for HPLC, ≥99.8%, contains 0.5%–1.0% ethanol as a stabilizer), *N,N*-dimethylformamide (anhydrous, 99.8%), Ethylene carbonate (98%), *p*-Toluenesulfonic acid monohydrate (ACS reagent, ≥98.5%), Pentaerythritol (for synthesis), Sodium bicarbonate (ReagentPlus®, ≥99.5%, crystalline), Anisole (anhydrous, 99.7%) and Vanillin (for synthesis) were purchased from Sigma Aldrich. Diethyl isophthalate (97%) was purchased from Syntree Inc. Dimethyl adipate (99%), Potassium carbonate (anhydrous, 99%), and Diethyl terephthalate (95%) were purchased from Alfa Aesar. 2-Propanol (ROTIPURAN®, >99.8%, p. a., ACS, ISO) and dimethyl sulfoxide (ROTIPURAN® ≥99,8%, p. a.) were purchased from Roth. Diethyl pyridine-2,4-dicarboxylate (PD24) was purchased from Carbosynth. Diethyl pyridine-2,5-dicarboxylate (PD24) was purchased from TCI.

Cygnet 2 and Cygnet 4 were synthesised according to a previous work (Warne et al., 2023).

### 2.2 Monomer synthesis

Compound **(1)** was synthesised according to a previously published procedure (Mankar et al., 2019), and was recovered as a white powder (35% yield).


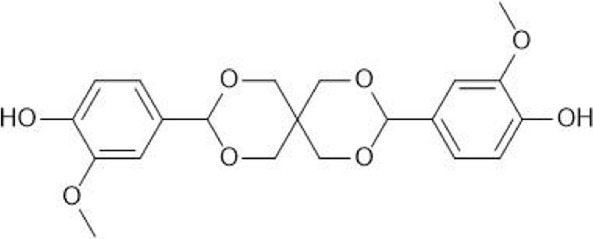


^1^H NMR (400 MHz, CDCl_3_, *δ*, ppm): 7.02 (d, J = 1.9 Hz, 2H), 6.96 (dd, J = 8.1, 1.9 Hz, 2H), 6.89 (d, J = 8.1 Hz, 2H), 5.66 (s, 2H), 5.39 (s, 2H), 4.89–4.81 (m, 2H), 3.90 (s, 6H), 3.87–3.76 (m, 4H), 3.63 (d, J = 11.6 Hz, 2H).

^13^C NMR (101 MHz, CDCl_3_, *δ*, ppm): 146.5, 146.4, 130.2, 119.6, 114.2, 108.3, 102.4, 71.2, 70.7, 56.0, 32.5.

HPLC-MS-ESI: retention time: 2.20 min (MS Spectrum: m/z 137.2); 7.57 min (MS Spectrum: m/z 271.2); 9.62 min (MS Spectrum: m/z 153.2).

Three different methods were used for the synthesis of compound **(2)**.

1. In dimethylformamide (DMF)

Compound **(2)** was first synthesised according to a previously published procedure (Mankar et al., 2019), and was recovered as a white powder (81% yield).

2. In dimethyl sulfoxide (DMSO)

This synthesis was adapted according to a previously published procedure (Mankar et al., 2019). Compound **(1)** (7.5 g, 18.6 mmol), ethylene carbonate (3.45 g, 39.2 mmol) and potassium carbonate (0.91 g, 6.6 mmol) were added to a 250 mL round-bottom flask with 50 mL DMSO. The reaction was heated from room temperature to 160 °C under N_2_, and stirred for 2 h. On completion of the reaction, it was left to cool down to room temperature, to which NaOH solution (25 mL, 0.1 N) was added dropwise. The mixture was vacuum filtered and washed with 1 L of water, resuspended in a second L of water, and finally vacuum filtered and washed with a third liter of water. Compound **(2)** was then lyophilised and was subsequently recovered as a white powder (84% yield).

3. Solventless, using excess ethylene carbonate (EC)

This synthesis was adapted according to a previously published procedure (Mankar et al., 2019). Compound **(1)** (3.74 g, 9.3 mmol), ethylene carbonate (as specified in the text) and potassium carbonate (0.456 g, 3.3 mmol) were added to a 100 mL round-bottom flask. The reaction was heated from room temperature to 160 °C under N_2_ and stirred for 2 h. On completion of the reaction, it was left to cool down to approx. 50 °C, to which NaOH solution (12.5 mL, 0.1 N) was added. The mixture was vacuum filtered and washed with water (250 mL). Compound **(2)** was dried under vacuum until a constant weight was obtained, yielding **(2)** as a white solid (80% yield).


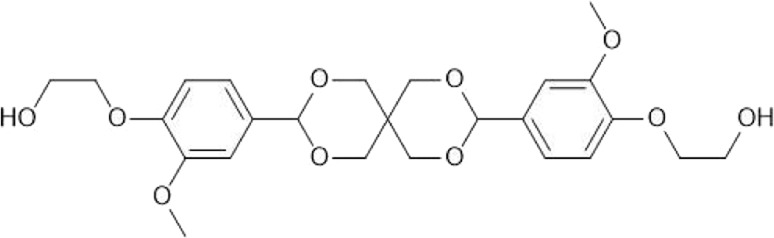


^1^H NMR (400 MHz, CDCl_3_, *δ*, ppm): 7.06 (d, *J* = 1.9 Hz, 2H), 7.02 (dd, *J* = 8.2, 1.9 Hz, 2H), 6.93 (d, *J* = 8.2 Hz, 2H), 5.42 (s, 2H), 4.87 (dd, *J* = 11.6, 2.5 Hz, 2H), 4.12 (dd, *J* = 5.3, 3.9 Hz, 4H), 3.92 (p, *J* = 4.5, 2.8 Hz, 4H), 3.89 (s, 6H), 3.84 (ddd, *J* = 11.5, 8.9, 2.0 Hz, 4H), 3.66 (d, *J* = 11.6 Hz, 2H), 2.59 (t, *J* = 6.3 Hz, 2H).

^13^C NMR (101 MHz, CDCl_3_, *δ*, ppm): 149.8, 148.7, 131.9, 119.0, 114.5, 109.5, 102.1, 71.5, 71.2, 70.7, 61.3, 55.9, 32.6.

HPLC-MS-ESI: retention time: 7.87 min (MS Spectrum: m/z 315.2); 9.32 min (MS Spectrum: m/z 197.2); 9.40 min (MS Spectrum: m/z 153.2); 12.27 min (MS Spectrum: m/z 493.4).

### 2.3 Polymer synthesis

8 x 10^−4^ mol of diester and an equimolar amount of **(2)** was added to a 25 mL round bottom flask with 2 g of solvent, and 10% by weight of monomers of CaLB (51.1–57.8 mg). The flask was heated to 85 °C and stirred at 400 rpm for 6 h, at which time the system was placed under vacuum (20 mbar) for a further 90 h. Reactions that used anisole as the reaction solvent were performed under reflux, and under N_2_ for the first 6 h.

Upon completion of the reaction, CHCl_3_ (unless otherwise specified) was added until the polymer was fully dissolved (5–25 mL), and the mixture was filtered through cotton to remove the enzyme. The flask was washed with approx. 1 mL of CHCl_3_ a further three times and all fractions were collected. The solvent was removed from the mixture via a rotary evaporator and transferred to a 50 mL Falcon tube containing ice cold methanol (35 mL) to precipitate the polymer. The mixture was vortexed and then centrifuged at 3,700 rpm for 10 min at 4 °C. The supernatant was removed, and 20 mL of ice-cold methanol was added, repeating the washing step a further two times.

### 2.4 Analytical techniques

#### 2.4.1 Nuclear magnetic resonance (NMR)

^1^H-NMR and ^13^C-NMR were performed in CDCl_3_ using a JEOL ECZ400R/S3 at a frequency of 400 MHz using tetramethylsilane (TMS, 0.03%) as a reference.

#### 2.4.2 High performance liquid chromatography-electrospray ionization-mass spectrometry (HPLC-ESI-MS)

All analyses were performed on an Agilent HPLC 1100. Samples were in CH_3_CN/H_2_O+ 0.1% FA 1:1 solution to make a final concentration of 600 μg/mL. A Gemini Hydro RP C18 150 × 3 mm column with a thickness of stationary phase of 4 μm was used. The temperature was set at 30 °C and the flow at 0.5 mL/min. The detector was a variable wavelength detector VWD with a l at 220 nm. The ionic source was an electrospray (ESI). The MS detector was a Microsaic 4,000 MiD single 53 Quadrupole, set to a Full scan in Positive mode with a TIC Voltage = 750 V and a Mass range of 100–800 m/z.

#### 2.4.3 Gel permeation chromatography (GPC)

Polymers were dissolved in CHCl_3_ to a concentration between 2 and 2.5 mg/mL and filtered through cotton wool packed into a 150 mm glass Pasteur pipette. The analysis was performed at 30 °C on an Agilent Technologies HPLC System (Agilent Technologies 1,260 Infinity) connected to a 17,369 6.0 mm ID × 40 mm LHHR-H, 5 μm Guard column and a 18,055 7.8 mm ID × 300 mm L GMHHR-N, 5 μm TSK gel liquid chromatography column (Tosoh Bioscience, Tessenderlo, Belgium) using CHCl_3_ as an eluent (at a flow rate of 1 mL/min for 20 min). An Agilent Technologies G1362A refractive index detector was employed for detection. Linear polystyrene calibration standards (250–70,000 Da) purchased from Sigma-Aldrich were used to calculate the molecular weights of the polymers.

## 3 Results & discussion

### 3.1 Synthesis and characterization of vanillin-derived monomer (2)

**SCHEME 1:**
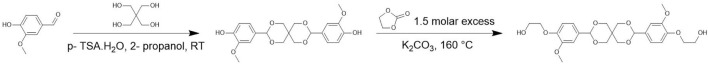
Two step synthesis of bio-based monomer **(2)** from vanillin. The first step from vanillin to **(1)** was performed unchanged from the work of Mankar et al. (2019), and the second step (monomer **(1)** to **(2)**) was performed without DMF, under the conditions shown.

Following the protocol reported in the literature, the diol monomer **(2)** was synthesised in two steps from vanillin (Mankar et al., 2019) (Scheme 1). The first double acetalization step with pentaerythritol was both non-hazardous and performed in a green solvent, therefore no changes were made to this procedure and structure **(1)** was obtained in 35% yield confirmed by NMR and HPLC-ESI-MS (Supplementary Figures S1–S3).

**FIGURE 2:**
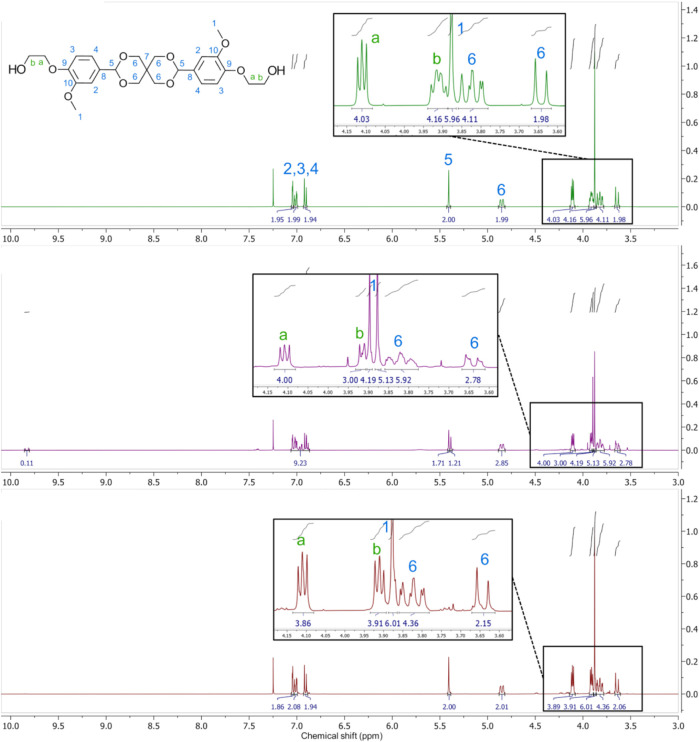
^1^H NMR spectra of the bio-based monomer (2) synthesised using: the initial procedure in DMF with peaks fully assigned (top), the solventless procedure using 0.15 M excess EC (middle), and the solventless procedure using 1.5 M excess EC (bottom). Region of interest (3.6–4.2 ppm) is enlarged for clarity.

The second step involving hydroxyethylation of **(1)** with ethylene carbonate (EC) was first performed in *N,N*-dimethylformamide (DMF) as per the original procedure (Mankar et al., 2019), obtaining a yield of 81% (NMR and HPLC-ESI-MS seen in Supplementary Figures S4–S6). DMF is considered a solvent of concern due to acute toxicity and reprotoxicity issues (N,N-Dimethylformamide anhydrous, 2024), and industrial use was recently restricted in the EU (Sherwood et al., 2024). Hence, dimethyl sulfoxide (DMSO) was selected as a greener replacement as it is also a dipolar aprotic with a similar boiling point. The reaction proceeded in DMSO, but there was a significant amount of residual solvent present in the final product. DMSO is known to induce unfolding of enzymes (Zhang, 2022a), so should be removed to avoid any negative effect on CaLB’s activity in the following polycondensation reaction. Despite washing **(2)** with several liters of water and performing multiple rounds of lyophilization, approximately 2% by weight of DMSO remained. As a result, we investigated the synthesis of **(2)** under solvent free conditions, as EC can act as both reagent and solvent due to its low melting point (35 °C–38 °C) (Kinage et al., 2008). **(2)** was synthesised under these conditions using different excesses of EC; 0.15 excess similar to the solventless procedure, and 1.5 M excess. ^1^H-NMR analysis of the products (Figure 2) shows the results of these syntheses compared with **(2)** synthesised in DMF (Figure 2A).

The use of a small excess of EC resulted in poor conversion, visible in Figure 2B. The ratio of peak a compared to peak 6 in Figure 2B. Indicates a conversion of around 70%, but it is likely that the partially hydroxyethylated compound will also be present, so the actual yield of **(2)** will be much lower. Comparing this to Figure 2C, **(2)** synthesised with 1.5 M excess EC has a much better conversion (>99%). Interestingly, further etherification of **(2)** was not observed despite the excess of EC, as no additional peaks were observed around 3.9 ppm. As a result, use of excess EC in place of DMF as a solvent can be considered a viable method to synthesise **(2)**. Unreacted EC could also potentially be recovered from the aqueous waste and reused, which would also decrease the toxicity of the waste stream.

### 3.2 Enzymatic polycondensation of (2) with various diesters

**FIGURE 3:**
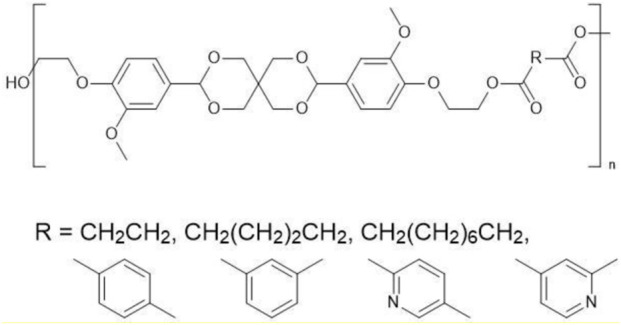
The chemical structure of the 7 polyesters produced in this work, synthesised from the diol **(2)**, and either: dimethyl succinate (DMSu), dimethyl adipate (DMA), dimethyl sebacate (DMSe), diethyl terephthalate (DET), diethyl isophthalate (DEI), diethyl pyridine-2,5-dicarboxylate (PD25) or diethyl pyridine-2,4-dicarboxylate (PD24).

**FIGURE 4:**
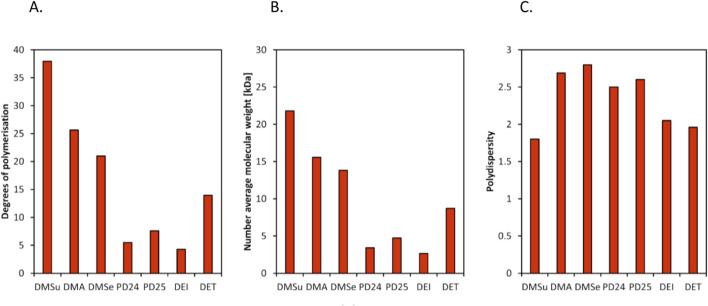
Results of enzymatic polycondensation of **(2)** with different diesters carried out in DPE, showing a comparison of **(A)** degrees of polymerisation (DP), **(B)** number average molecular weight (M_n_), and **(C)** polydispersity (Đ).

Previous polymer synthesis reactions with **(2)** have focused on the production of co-polymers (Mankar et al., 2019), illustrating the different mechanical and thermal properties that can be achieved with different amounts of **(2)** incorporated into the polymer. This work investigates the use of enzymes in this reaction, focusing on the synthesis of homopolymers and the effect that various diesters have on the molecular weight. Enzymatic polycondensations of **(2)** were carried out with both aliphatic and aromatic diesters for a total of 7 different polyesters (Figure 3). The aliphatic diesters of acids with a chain length from C4 to C10 including dimethyl succinate (DMSu), dimethyl adipate (DMA), and dimethyl sebacate (DMSe), and the aromatic monomers diethyl terephthalate (DET), diethyl isophthalate (DEI), diethyl pyridine-2,5-dicarboxylate (PD25) and diethyl pyridine-2,4-dicarboxylate (PD24) were used. Initial reactions were performed in diphenyl ether (DPE), an aprotic solvent commonly used in literature polycondensations catalysed by CaLB (Mahapatro et al., 2003; Pellis et al., 2020). The polymerization results can be seen in Table 1 and Figure 4 and the SI (Supplementary Figures S7).

**TABLE 1:** Molecular weight results of the enzymatic polycondensation of vanillin-based monomer **(2)** with different diesters carried out in DPE, showing structure, number average (M_n_) and weight average molecular weight (M_w_), dispersity (Đ) and degrees of polymerization (DP).

Diester	Structure	M_n_ [kDa]	M_w_ [kDa]	Đ	DP
DMSu	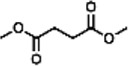	21.8	39.2	1.80	37.9
DMA	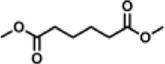	15.6	41.8	2.69	25.6
DMSe	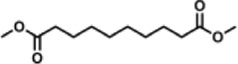	13.8	38.8	2.80	21.0
PD24	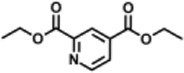	3.4	8.6	2.5	5.5
PD25	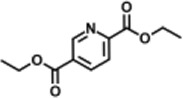	4.7	12.3	2.6	7.6
DEI	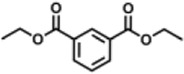	2.7	5.5	2.05	4.3
DET	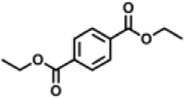	8.7	17.1	1.96	14.0

Interestingly, the DMSu-**(2)** polymer had the highest number average molecular weight observed in this work, at 21.8 kDa (M_n_). For polymers based on aliphatic diesters, there was a trend of increased M_n_ with decreased chain length of the diester. M_w_ however, was extremely similar, showing that these three aliphatic polyesters differ mainly in terms of molecular weight distribution, seen in Figure 4C. There is a precedent for use of DMA leading to higher molecular weight polymers when compared to DMSe (Pellis et al., 2018), but the DMSu based polymer having such a high molecular weight is unusual. However this previous work has a significant difference; reactions were performed under solventless conditions (Pellis et al., 2018), which has been shown to affect molecular weight (Mahapatro et al., 2003). For polycondensations conducted in solvent, solubility of both monomers as well as the polymer must be considered. It is possible that the DMSu-**(2)** polymer is particularly soluble in DPE, resulting in higher molecular weights. The increased reactivity of shorter chain diesters may also be a factor, as it has been noted in previous literature that the rate of acid catalysed esterification reactions decreased with increasing chain length (Liu et al., 2006). Steric hindrance was a notable factor in this previous work, which would also apply here.

For those polycondensations with two aromatic/semi-aromatic monomers, molecular weights are much lower, as expected. There are several examples in the literature of CaLB catalysed polycondensations utilizing aromatic monomers only (Komarova et al., 2024; Pellis et al., 2016; 2019; Salum et al., 2024), many of which resulted in low molecular weight polymers (M_n_ < 4,000 Da) (Pellis et al., 2016; Salum et al., 2024). This is generally attributed to the low reactivity of such monomers under the reaction conditions used, or poor solubility of the semi-aromatic polymers in the reaction media (Douka et al., 2018). Here an aromatic diester (either PD24, PD25, DEI or DET) was polymerised with a semi aromatic diol (monomer **(2)**), so this effect should be even more pronounced. It is important to highlight how solventless conditions are unsuitable for polycondensation of aromatic monomers as they often have high (>100 °C) melting points to be used in enzymatic reactions.

Another trend of note is that use of *para-*substituted diesters yielded higher molecular weight polymers compared to *meta-*substituted diesters, for both phthalic and pyridinedicarboxylic acid-based structures (Figure 4B). For PD24 and PD25, the opposite was observed when these monomers were polymerised with aliphatic diols (Komarova et al., 2024; Pellis et al., 2019). This was thought to be due to a difference in solubility of the synthesised polymers, so it is possible that PD25-**(2)** polyesters are simply more soluble in DPE compared to PD24-**(2)** polyesters. The same is true of DEI and DET based polyesters, as a previous work attributed low molecular weights of synthesised poly (1,6-hexanediol terephthalate) compared to poly (1,6-hexanediol isophthalate) to poor solubility of the former in the reaction media (in this case toluene) (Mezoul et al., 1996).

Overall, molecular weights were high, but the limiting factor seemed to be solubility of the polymers in DPE. By 96 h the reaction mixes for the DMA, DMSu and DMSe based polymers were highly viscous, hindering stirring, but it is possible that reaction solvent volume is a parameter that can be further optimised to further increase molecular weights. However, another issue was the precipitation of DPE together with the polymer in methanol (MeOH), during the workup stage despite their miscibility, which further complicates the workup.

Despite this, enzymatic polycondensations may be more suitable for producing polyesters based on **(2)** when compared to chemical catalysis. In a previous work, synthesis of polyesters from **(2)** and dimethyl terephthalate using a dibutyltin oxide (DBTO) catalyst resulted in polyesters with a M_n_ of 1.2 kDa (Mankar et al., 2019), whereas here a M_n_ of up to 8.7 was achieved for the same polymer. Of course, it must be taken into consideration that enzymatic polycondensations are run for much longer; 96 h in this work compared to 16 h in the work of Mankar et al. (2019). The low molecular weight in the DBTO catalysed polycondensation was attributed to steric hindrance from the bulky **(2)** monomer affecting esterification when conversion approached 100%.

### 3.3 Investigating green solvents

Although DPE has been proven to be an excellent solvent for this reaction, it is also highly environmentally toxic and derived from petroleum. In addition, DPE contamination in the final polymer is significant, despite multiple washing steps (Supplementary Figures S8–S14). Due to these reasons, efforts were made to replace DPE as the reaction solvent, and several alternatives were tested in the polycondensation of DMA and **(2)**.

The initial choice was Cyrene and other Cygnet derivatives, as solvents that are both bio-based and have previously been shown to be very suited for enzymatic polycondensation reactions (Warne et al., 2023). However, initial tests showed that **(2)** had limited solubility in Cyrene, Cygnet 2 and Cygnet 4, and indeed, polycondensations in these media resulted in low molecular weights (Table 2). Instead, anisole was selected as a solvent analogous to DPE; due to its lower boiling point it can be removed more readily during workup, and unlike DPE it is not an environmental hazard. Anisole has also been successfully used as reaction media for enzymatic polycondensations in the literature (Salum et al., 2024).

**TABLE 2:** Results of initial enzymatic polycondensation of DMA and vanillin-based **(2)** carried out in different solvents.

Reaction solvent	Observations^a^	Conversion [%]^b^	M_n_ [kDa]	M_w_ [kDa]	Đ	DP^c^
Cyrene	Limited solubility of **(2)**	98	3.3	13.8	4.20	5.4
Cygnet 2	Limited solubility of **(2)**	92	1.4	5.9	4.23	2.3
Cygnet 4	Limited solubility of **(2)**	58	0.4	1.0	2.35	0.7
DPE	Hindered stirring at 96 h	>99	15.6	41.8	2.69	25.6
Anisole	Hindered stirring at 96 h and evaporation of anisole	>99	21.0	45.5	2.17	34.6

^a^ Visual observations during synthesis.

^b^ calculated from ^1^H-NMR.

^c^ Degree of polymerisation.

Minor changes were required in the procedure; due to the lower boiling point and hygroscopicity, the reaction was conducted under reflux and under N_2_ for the first 6 h. A large proportion of anisole was lost to evaporation over the course of the reaction but still resulted in a polymer with a M_n_ of 21 kDa (Table 2). Slow evaporation of the anisole over 96 h would concentrate the monomers, possibly increasing mass transfer and therefore reaction rate.

Although the reaction of DMA and **(2)** in anisole was initially promising, it was repeated several times and the issue of reproducibility in terms of molecular weights can clearly be seen in Table 3. Although the initial polycondensation resulted in a polyester with a M_n_ of 21 kDa, subsequent polycondensations led to polymers having much lower molecular weights.

**TABLE 3:** Repeat reactions of the enzymatic polycondensation of DMA and vanillin-based (2) carried out in anisole. Average is calculated with standard deviation in brackets.

Reaction	Conversion [%]^a^	M_n_ [kDa]	M_w_ [kDa]	Đ	DP^b^
1	>99	21.0	45.5	2.17	34.6
2	81	4.6	6.8	1.47	7.6
3	>99	7.0	14.4	2.05	11.6
Average	94 (+/-9)	11.3 (+/-7.7)	22.0 (+/-16.5)	1.85 (+/-0.27)	18.6 (+/-12.8)

^a^ calculated from ^1^H NMR.

^b^ degree of polymerisation.

The variability in molecular weights is considerable and is reflected in the large standard deviation in the M_n_, M_w_ and DP values. There is some variation in conversion, but despite reactions 1 and 3 both having a conversion >99%, molecular weights are still very different. Here, although the initial transesterification of **(2)** and DMA is similar in the early stage of the reaction and almost all of **(2)** reacts, the subsequent chain elongations proceed very differently. The reason for this is likely due to the used reaction medium. As previously mentioned, anisole is hygroscopic and even a small amount of water present may result in the hydrolysis of the polymer chains. Additionally, use of a reflux condenser means any water present is removed more slowly. Notably, the first synthesis performed in anisole from the freshly opened bottle (that is, fully anhydrous) gave the highest molecular weight by far (21.0 kDa M_n_). Another potential cause is the reflux condensers used. Several different condensers were used, which had a noticeable effect on the evaporation rate of anisole. Use of waterless condensers resulted in extremely fast evaporation of anisole, and the matrix had visibly hindered stirring at a much earlier timepoint compared to those reactions run with conventional condensers. Faster evaporation of anisole could reduce mass transfer and significantly reduce the final molecular weights. The inconsistency in molecular weights of polyesters synthesised in anisole is likely to be due to both factors, the presence of water and variability in the evaporation rate of anisole.

Although there are more factors to consider when using anisole as a reaction solvent, the difficulties mentioned above are not insurmountable. To ensure anisole is completely anhydrous it should be kept under an inert atmosphere and in the presence of molecular sieves. An efficient condenser should also be used to minimize anisole evaporation over the course of the reaction. Finally, vacuum strength is a key parameter that should be further investigated for this reaction; ideally it would be sufficient to readily remove the MeOH byproduct whilst keeping all the low boiling organic solvent in the reaction flask.

Overall, although anisole showed some promise in enzymatic polycondensation reactions, under the reaction conditions used in this work, it is unsuitable for consistently producing high molecular weight polymers. This is mainly due to its lower boiling point and hygroscopicity, but the reaction conditions can be further optimised to minimize solvent loss, as well as maintain an anhydrous system. Although more suitable, polycondensations in DPE are not without their issues as the main problem with this synthesis is the precipitation of DPE together with the polymer during the workup stage, despite its solubility in methanol. Anisole was initially selected for its similarity to DPE but there are other potential solvent candidates that may have similar effects without the need of an anhydrous system, such as ethoxybenzene, propoxybenzene or tert-butoxybenzene. However, Salum *et al.* compared anisole and ethoxybenzene (among others) in the enzymatic polycondensation of PD24 and 1,8-octanediol, and found that use of anisole resulted in higher M_n_ (Salum et al., 2024). Yet these reactions were conducted under 360 mbar vacuum, meaning the methanol by-product is removed less rapidly, and for a shorter time. A systematic investigation of enzymatic polycondensations in lower bp media is needed with regards to vacuum strength, reaction time, and temperature, to fully exploit these solvents.

## 4 Conclusion

Overall, a series of polyesters based on the vanillin-derived diol **(2)** were successfully enzymatically synthesised. Attempts to replace DMF solvent in the synthesis of monomer **(2)** were successful; although initial trials in DMSO worked well, it was difficult to remove, but use of 1.5 M excess EC resulted in good conversion (>99%). To further increase the sustainability of this reaction, efforts should be made towards recovering pure EC from the waste and reusing it in subsequent syntheses.

Finding a suitable green solvent for the enzymatic polycondensation reactions of **(2)** was more difficult. Despite the initial success of anisole in producing high molecular weight polymers, results were inconsistent, and when **(2)** was polymerised with aromatic diesters, DPE was the superior reaction media by far. However, use of anhydrous anisole and a reaction system optimised to minimize solvent evaporation has the potential to consistently produce vanillin-based polyesters with high molecular weights. In addition, the fact that CaLB can be used to polymerise bulky semi aromatic monomers such as **(2)** cannot be ignored. Although the polycondensation of **(2)** with aliphatic diesters yielded higher molecular weights (with the DMSu based polyester reaching up to 21.8 kDa M_n_), the molecular weights of polyesters based on aromatic diesters are also significant (8.7 kDa M_n_ for the polycondensation of **(2)** and DET). In this work we have demonstrated the versatility of CaLB in producing aliphatic-aromatic and aromatic polyesters from various bio-based sources in both high boiling and medium boiling organic media, both of which are able to preserve enzymatic activity over several days of reaction.

## Data Availability

The raw data supporting the conclusions of this article will be made available by the authors, without undue reservation.
