# Supplementary material for: Enzymatic synthesis of bio-based polyesters derived from vanillin
Source: Front Chem. 2026 Feb 20;14:1769290. doi: 10.3389/fchem.2026.1769290 (PMC12962657; doi:10.3389/fchem.2026.1769290)
Supplement: Supplementary file 1 [file DataSheet1.docx]

Figures


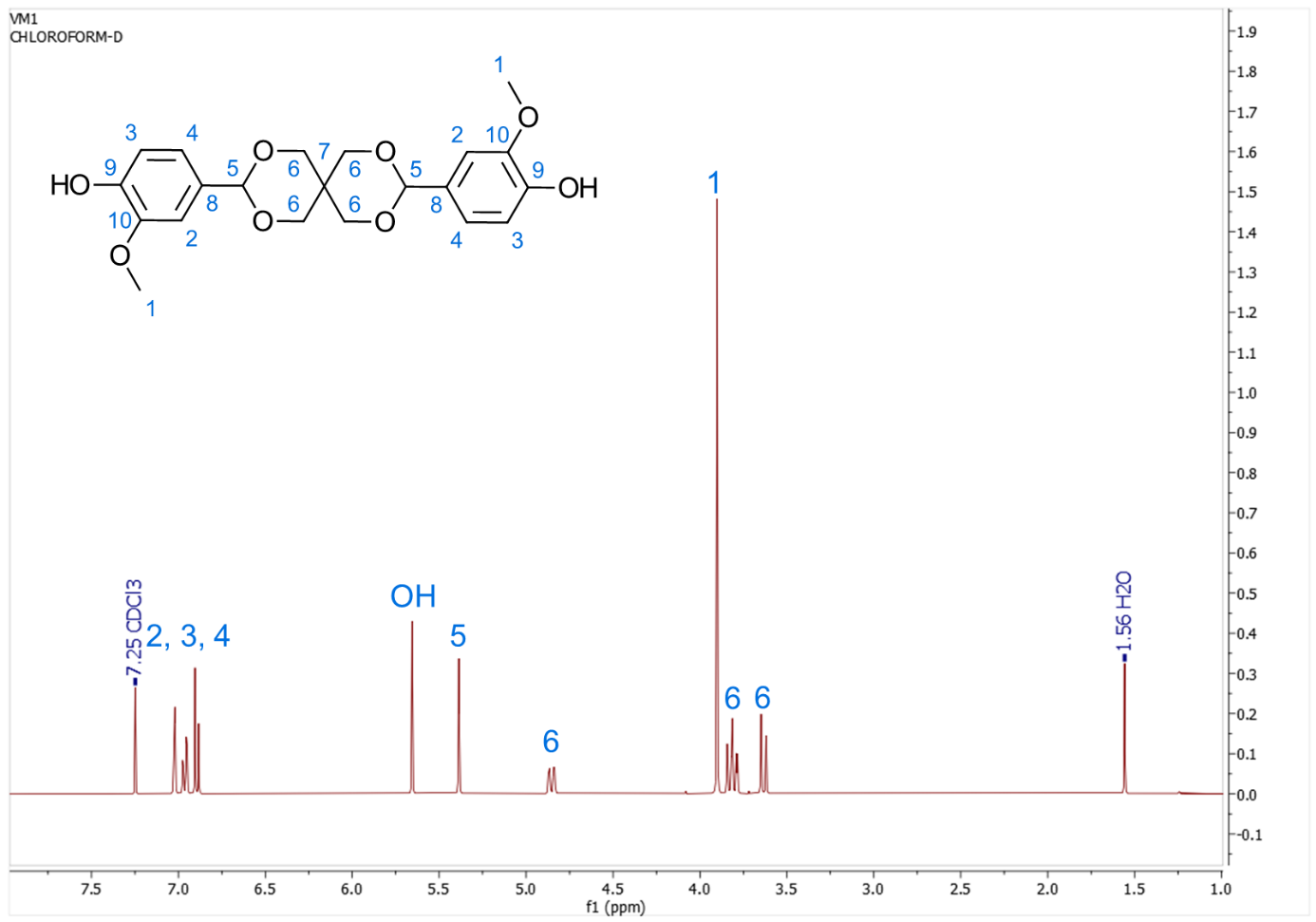


*Figure S 1. ^1^H NMR spectra and chemical structure of monomer* ***(1)****, with all peaks assigned.*


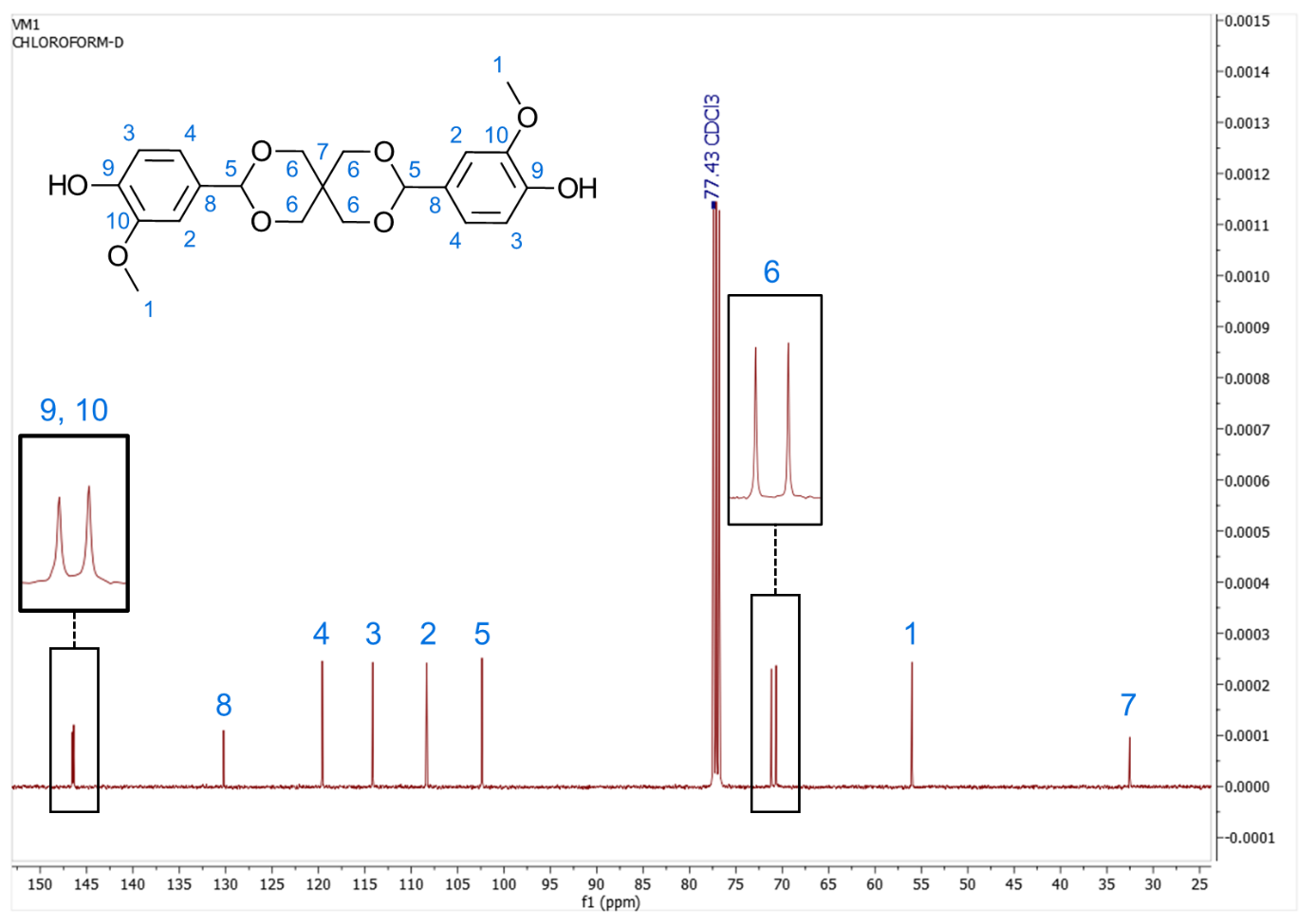


*Figure S 2. ^13^C NMR spectra and chemical structure of monomer* ***(1)****, with all peaks assigned. Peaks from 68 – 75 ppm are magnified.*


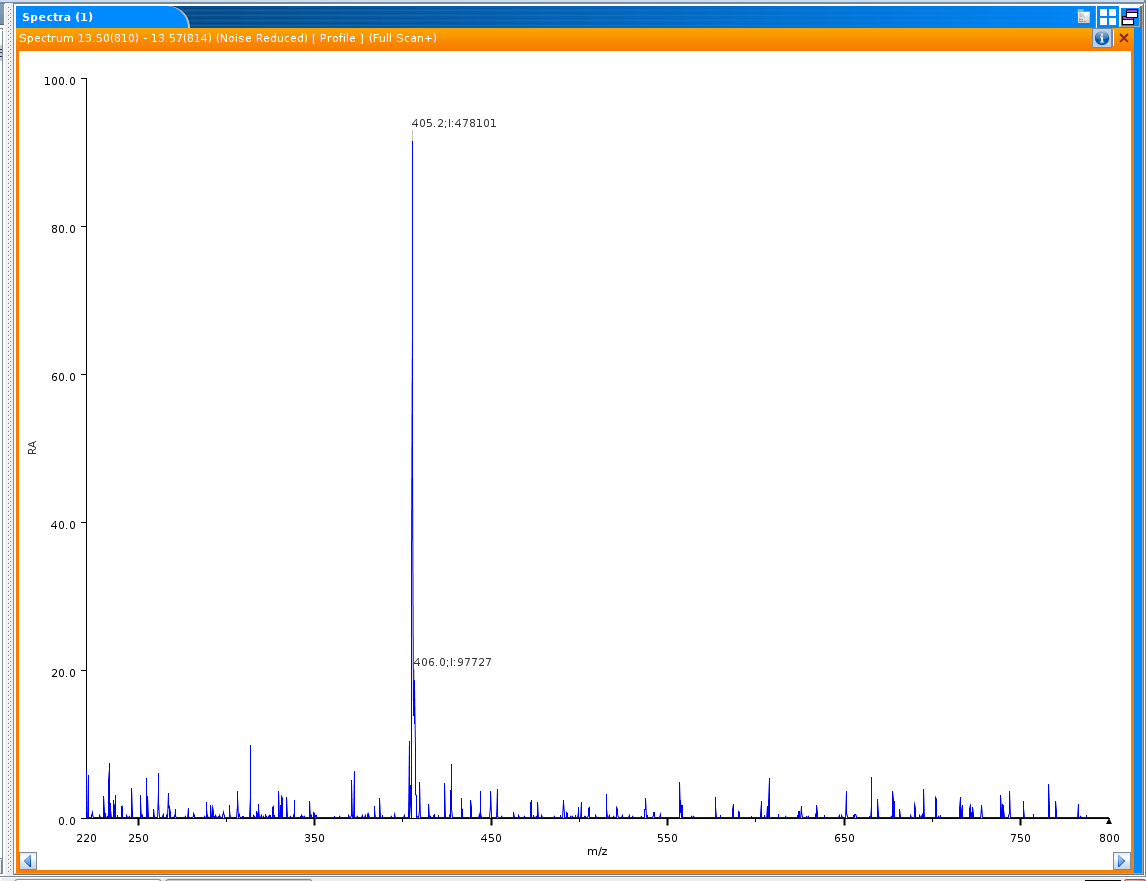


*Figure S 3. ESI-MS spectra of monomer* ***(1)****, showing the mononuclear ion with an observed mass of 405.2.*


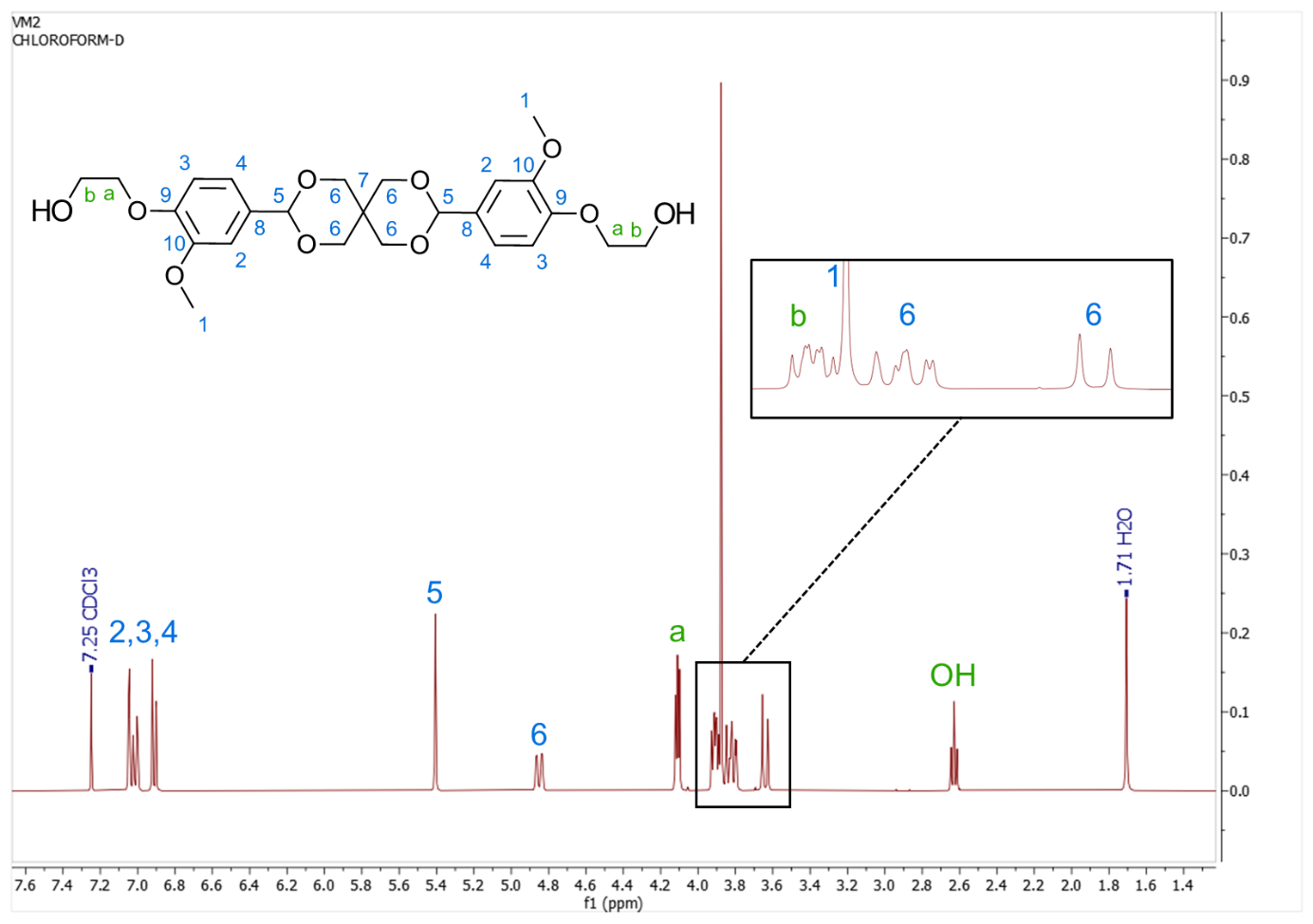


*Figure S 4. ^1^H NMR spectra and chemical structure of monomer* ***(2)****, with all peaks assigned. Peaks from 3.5 – 4.0 ppm are magnified.*


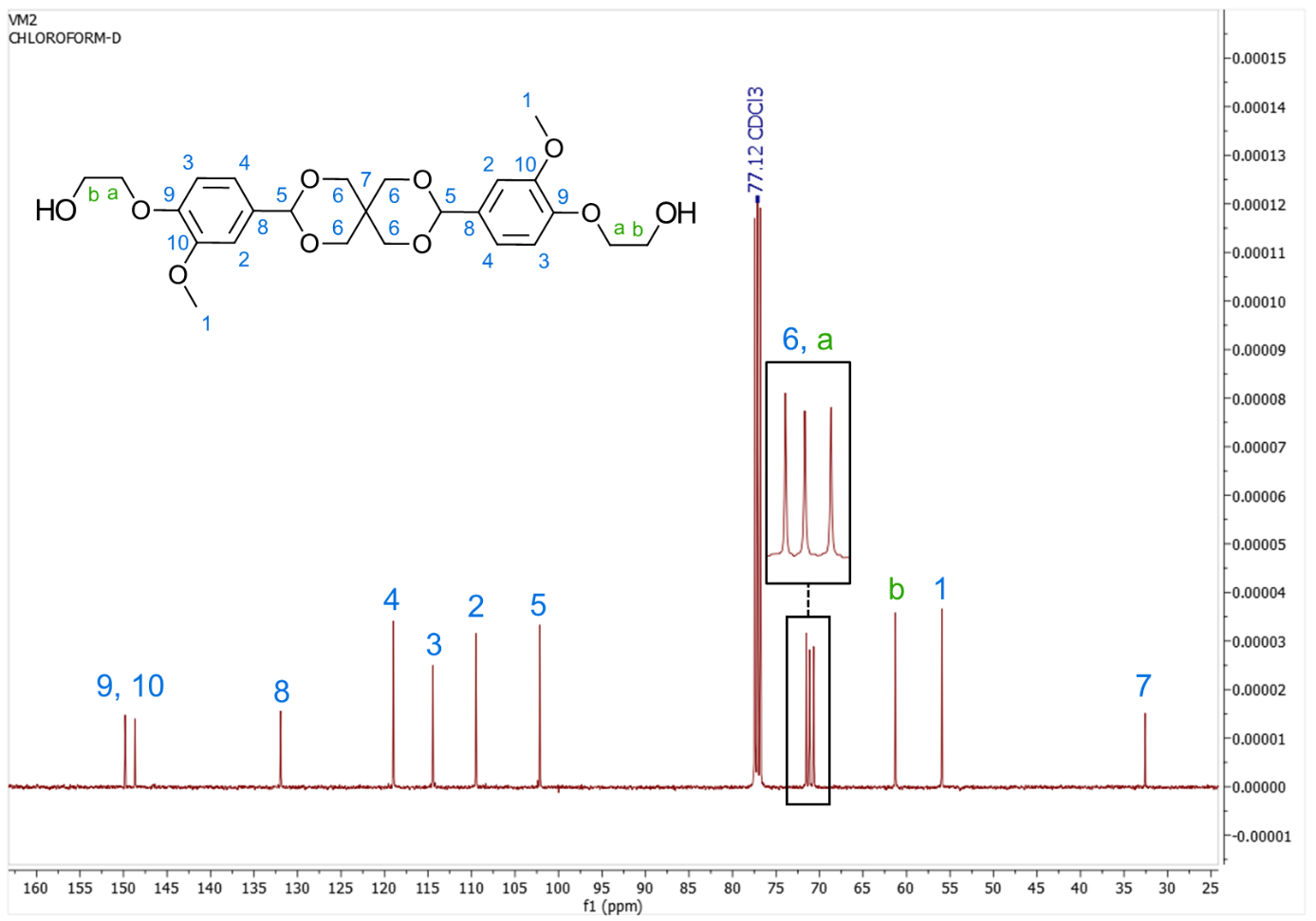


*Figure S 5. ^13^C NMR spectra and chemical structure of monomer* ***(2)****, with all peaks assigned. Peaks from 68 – 75 ppm are magnified.*


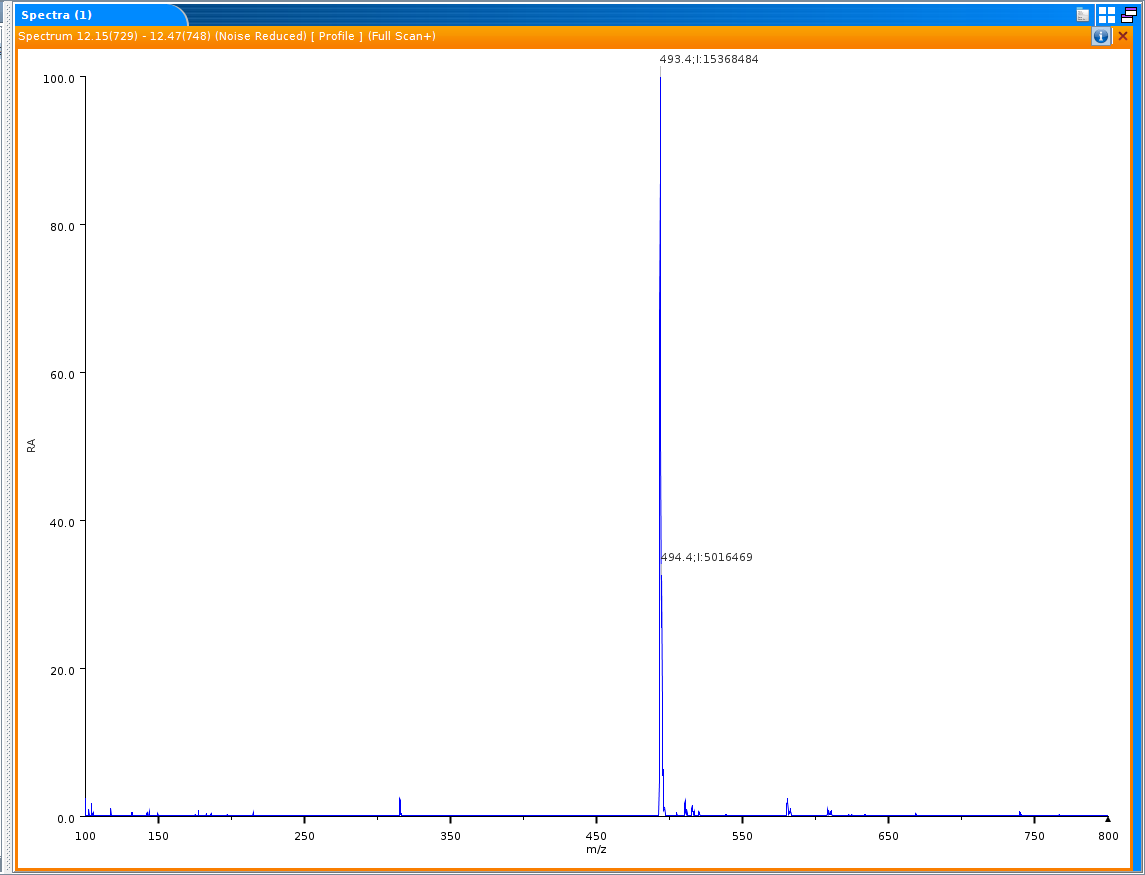


*Figure S 6. ESI-MS spectra of monomer* ***(2)****, showing the mononuclear ion with an observed mass of 493.4.*


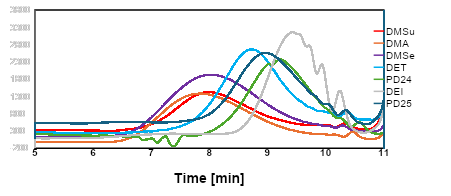


*Figure S 7. GPC spectra of the seven different polyesters based on monomer (2) synthesised in DPE (close up of the region from 5 – 11 min). Polyester structures are denoted by the structure of the diester unit (DMSu, DMA, DMSe, DET, PD24, DEI, PD25).*

*
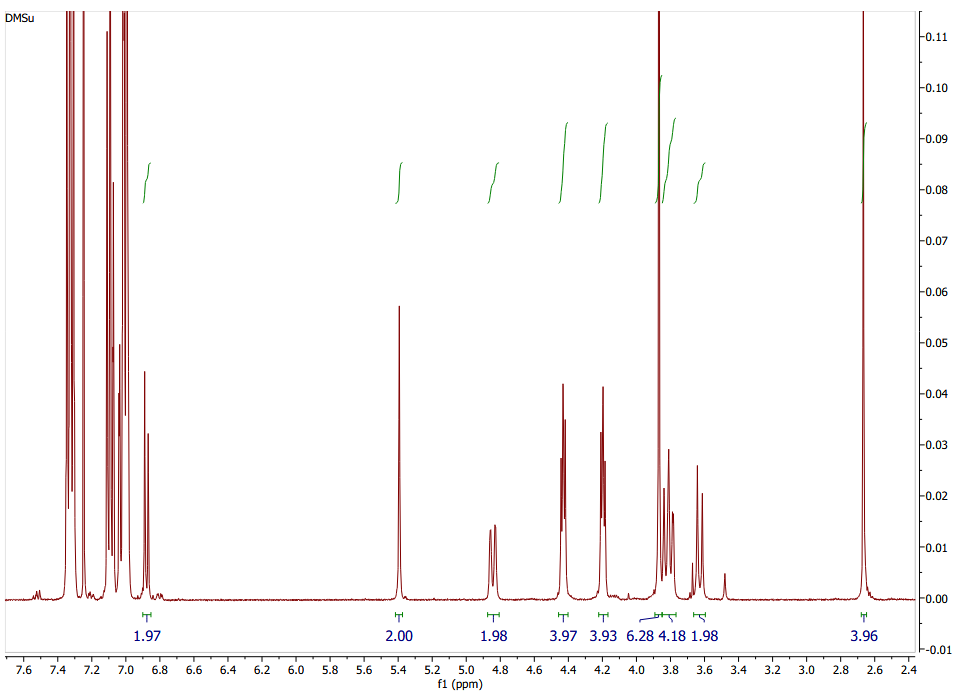
Figure S 8. ^1^H NMR spectra of the VM2-DMSu polymer synthesised in DPE.*

*
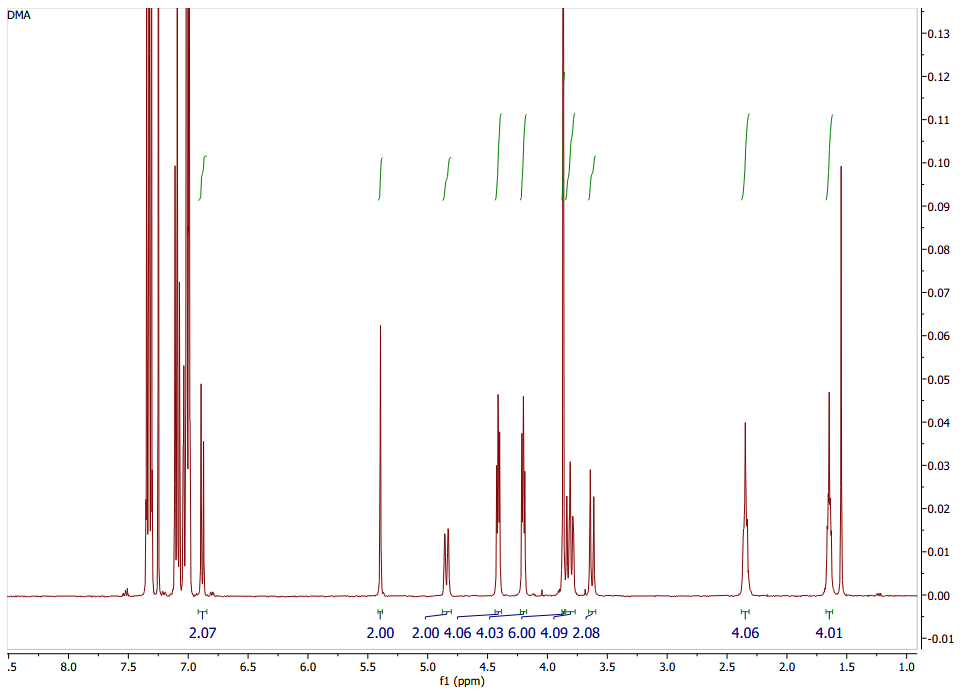
Figure S 9. ^1^H NMR spectra of the VM2-DMA polymer synthesised in DPE.*

*
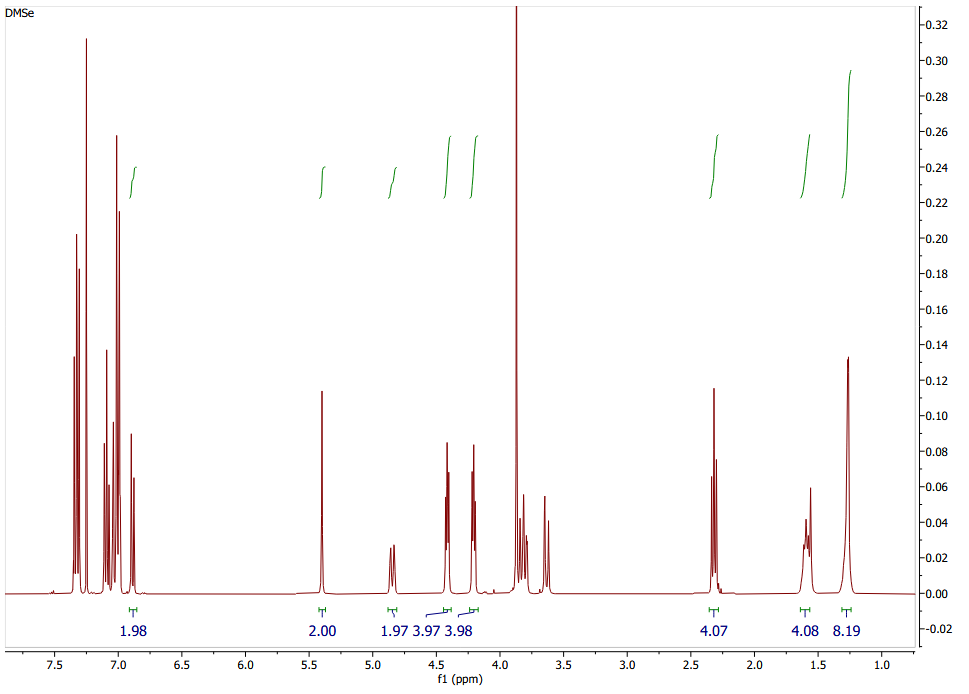
Figure S 10. ^1^H NMR spectra of the VM2-DMSe polymer synthesised in DPE.*

*
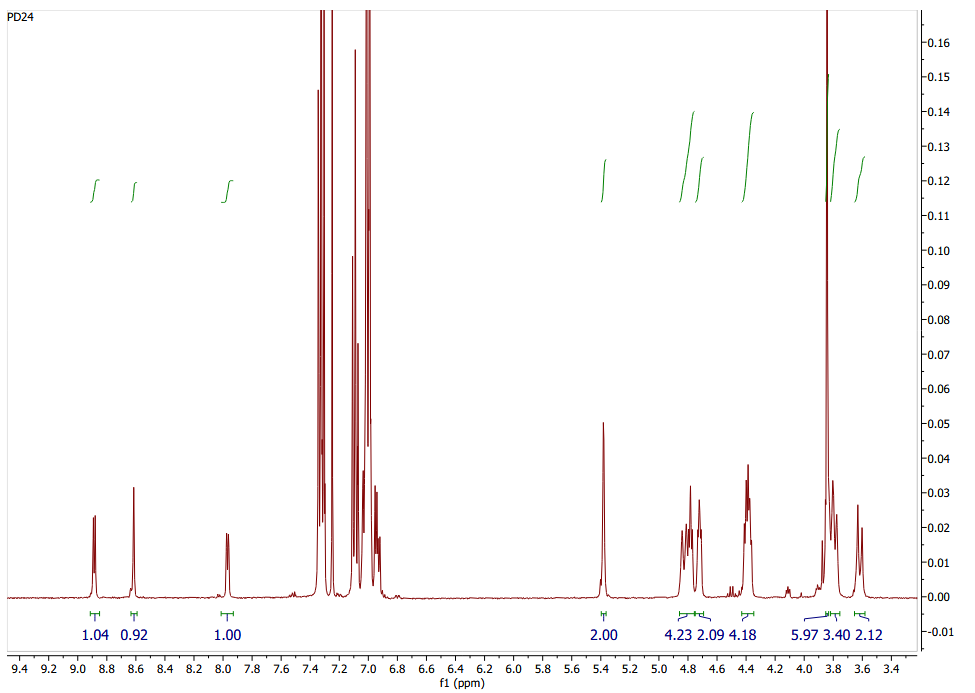
*

*Figure S 11. ^1^H NMR spectra of the VM2-PD24 polymer synthesised in DPE.*

*
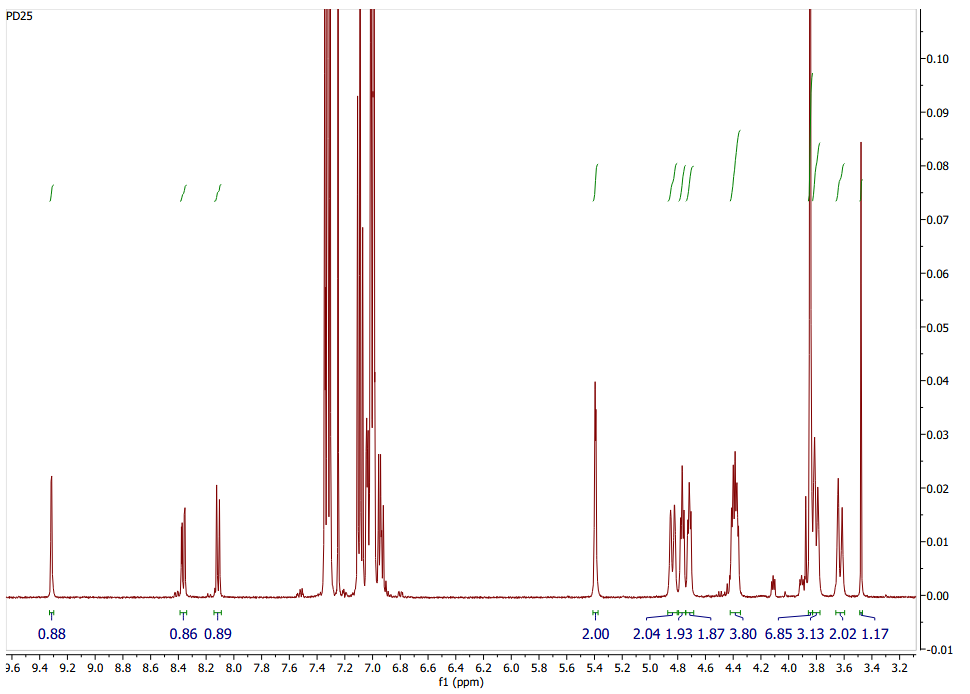
*

*Figure S 12. ^1^H NMR spectra of the VM2-PD25 polymer synthesised in DPE.*

*
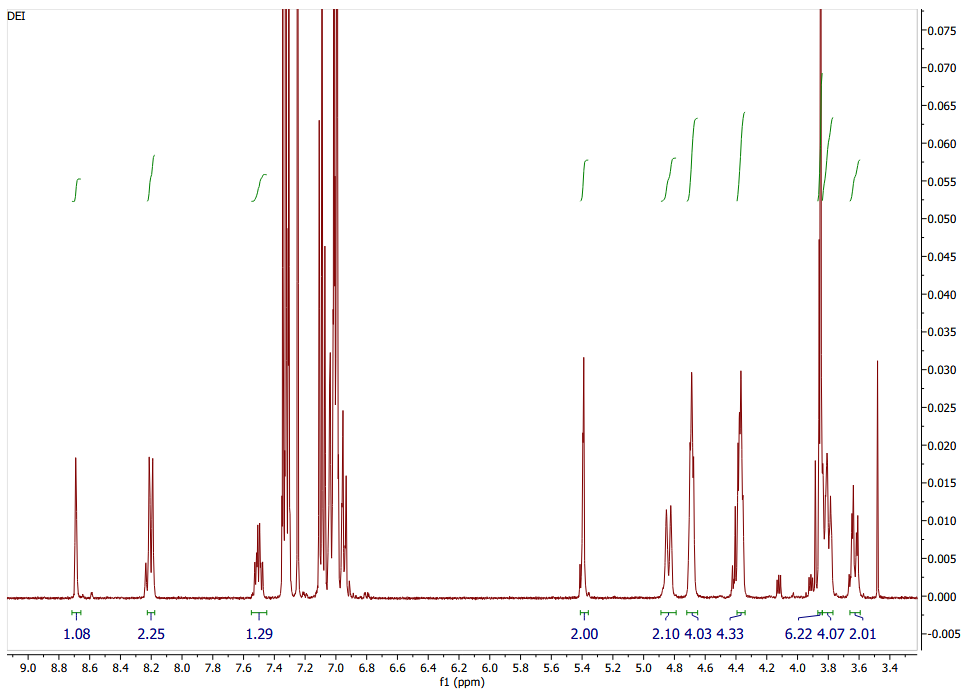
*

*Figure S 13. ^1^H NMR spectra of the VM2-DEI polymer synthesised in DPE.*

*
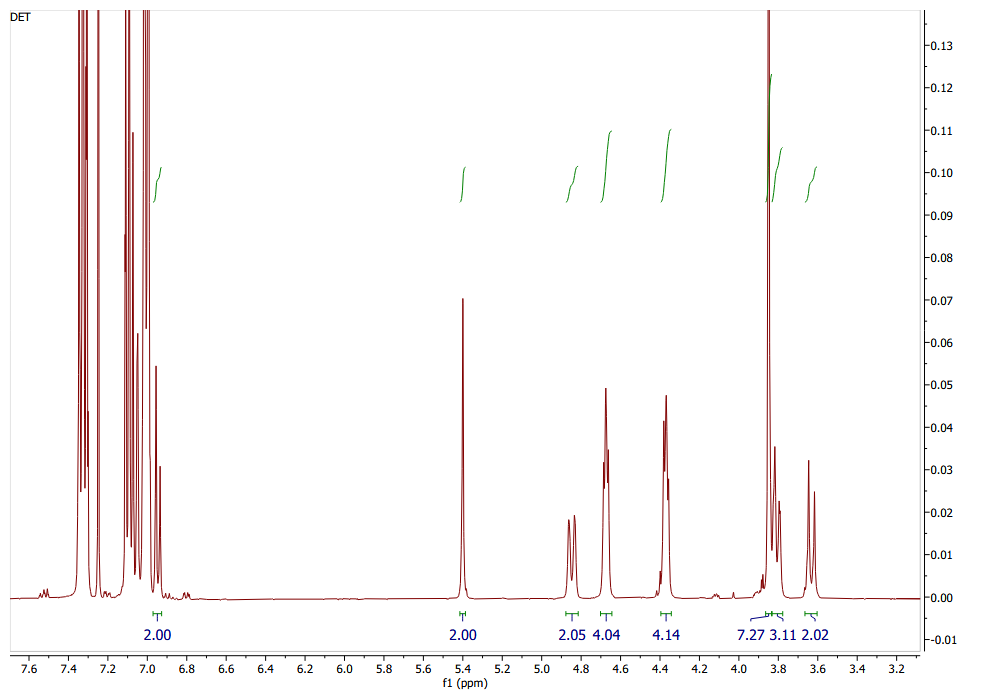
*

*Figure S 14. ^1^H NMR spectra of the VM2-DET polymer synthesised in DPE.*
